# Passively Mode-Locked Er-Doped Fiber Laser Based on Sb_2_S_3_-PVA Saturable Absorber

**DOI:** 10.3390/molecules27030745

**Published:** 2022-01-24

**Authors:** Qiongyu Hu, Xiaohan Chen, Ming Li, Ping Li, Liwei Xu, Haoxu Zhao, Bin Zhang, Jing Liu, Kejian Yang

**Affiliations:** 1 Center for Optics Research and Engineering, Key Laboratory of Laser & Infrared System, Ministry of Education, Shandong University, Qingdao 266237, China; 201920407@mail.sdu.edu.cn (Q.H.); 201933705@mail.sdu.edu.cn (L.X.); bin-zhang@mail.sdu.edu.cn (B.Z.); 2 School of Information Science and Engineering, Shandong Provincial Key Laboratory of Laser Technology and Application, Shandong University, Qingdao 266237, China; liming1982@sdu.edu.cn (M.L.); pingli@sdu.edu.cn (P.L.); 202112661@mail.sdu.edu.cn (H.Z.); 202012720@mail.sdu.edu.cn (J.L.); 3 Institute of Novel Semiconductors, State Key Laboratory of Crystal Materials, Shandong University, Jinan 250100, China

**Keywords:** antimony trisulfide, fiber laser, mode-locked, saturable absorber

## Abstract

In this paper, antimony trisulfide (Sb_2_S_3_) was successfully prepared with the liquid phase exfoliation method and embedded into polyvinyl alcohol (PVA) as a saturable absorber (SA) in a passively mode-locked Er-doped fiber laser for the first time. Based on Sb_2_S_3_-PVA SA with a modulation depth of 4.0% and a saturable intensity of 1.545 GW/cm^2^, a maximum average output power of 3.04 mW and maximum peak power of 325.6 W for the stable mode-locked pulses was achieved with slope a efficiency of 0.87% and maximum single pulse energy of 0.81 nJ at a repetition rate of 3.47 MHz under a pump power of 369 mW. A minimum pulse width value of 2.4 ps with a variation range less than 0.1 ps, and a maximum signal to noise ratio (SNR) of 54.3 dB indicated reliable stability of mode-locking, revealing promising potentials of Sb_2_S_3_ as a saturable absorber in ultrafast all-fiber lasers.

## 1. Introduction

In past decades, ultrafast pulsed all-fiber lasers have been extensively applied in various fields from industrial applications to fundamental research including material processing, optical devices, optical communications, fiber sensors, laser radar, microscopy imaging, medical treatment, etc., [1,2,3,4,5] Q-switching and mode-locking techniques were the most important and most common methods to achieve ultrafast pulses in fiber lasers [6,7]. Compared with Q-switching, mode-locking attracted more considerable attention because of narrower pulse width and higher peak power. Recently, various kinds of two-dimensional (2D) materials, such as graphene [8,9], graphene oxide (GO) [10], MXenes [11], single-walled carbon nanotubes (SWCNTs) [12], quantum wells (QWs) [13], black phosphorus (BP) [14], topological insulators (TIs) [15,16,17], transition metal dichalcogenides (TMDCs) [18,19,20,21,22,23,24], and layered metal sulfides (LMSs) [25] were widely used as saturable absorbers in mode-locked fiber lasers due to their outstanding nonlinear saturable absorption properties caused by sub-band gap absorption or the Pauli blocking principle [26]. In particular, various TMDCs including MoS_2_ [19] and WS_2_ [21], etc. have been extensively used as a mode-locker for achieving mode-locked Er-doped fiber lasers due to lamellar-dependent band gaps [27] and readily exfoliative layered structures with strong in-plane bonding and weak out-of-plane interactions [28,29]. What’s more, LMSs such as ReS_2_ [30] and SnS_2_ [31,32], etc. have held certain attention in mode-locked fiber lasers owning to layer-dependent structural, electronic, and optical properties [25]. In 2017, Zhao et al. reported a passively Q-switched and mode-locked Er-doped fiber laser based on an ReS_2_ saturable absorber [30]. Soon after, passively mode-locked operations based on SnS_2_ SA in both Yb-doped and Er-doped fiber lasers were achieved by Zhang et al. [32,33].

Antimony sulfide (Sb_2_S_3_) was a novel LMSs that possessed an orthorhombic structure consisting of ribbonlike chains of parallel molecular (Sb_4_S_6_)_n_ [33]. And there was a direct bandgap that belonged to Sb_2_S_3_ that could be tuned between 1.5–2.3 eV by changing the size, shape and crystallinity [34], which was appropriate for visible and infrared emissions. Sb_2_S_3_ has been widely demonstrated in many fields such as photocatalyst, solar cells, photonic integrated circuits, etc., [35,36,37,38], due to its outstanding physical and optical properties including exotic photoconductive effect, high Seebeck coefficient, high absorption coefficient, etc., [34,37]. However, there were few attentions paid to study on nonlinear optical properties of Sb_2_S_3_. In 2021, Zhou reported a 1.06 μm passively Q-switched solid-state laser based on Sb_2_S_3_ SA with peak power as high as 2.68 W [39]. Nevertheless, there were no reports on Q-switched or mode-locked fiber lasers based on Sb_2_S_3_ SA yet. 

In this paper, Sb_2_S_3_-PVA SA was successfully prepared and employed as a nonlinear SA for demonstrating a passively mode-locked Er-doped all-fiber laser. It was based on a film-type SA, which was composed of highly crystalline and pure Sb_2_S_3_ nanosheets prepared by the liquid phase exfoliation method and polyvinyl alcohol (PVA) matrix. Microscopic properties of Sb_2_S_3_ were deeply researched, and the nonlinear character of Sb_2_S_3_-PVA SA was measured by a power-dependent transmission scheme, demonstrating that modulation depth, saturable intensity and non-saturable absorbance were 4.0%, 1.545 GW/cm^2^ and 46%, respectively. Stable mode-locked operations were realized under pump power from 75.1 to 369 mW at repetition rate of 3.74 MHz. A maximum average output power of 3.04 mW and a maximum peak power of 325.6 W for the stable mode-locked pulses were achieved with a slope efficiency of 0.87% and maximum single pulse energy of 0.81 nJ at a pump power of 369 mW. The signal to noise ratio (SNR) always exceeded 45 dB at any pump power, and there was a minimum pulse width of 2.4 ps with a variation range less than 0.1 ps, revealing fairly stable mode-locked operations in this all-fiber laser.

## 2. Preparation and Characterization of Sb_2_S_3_

### 2.1. Preparation of Sb*_2_*S*_3_*-PVA SA

**Figure 1:**
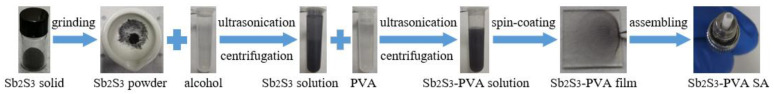
Preparation flow diagram of Sb_2_S_3_-PVA SA.

The Sb_2_S_3_ nanosheets were successfully prepared with the liquid phase exfoliation method by using ultrasonic cleavage. Subsequently, few-layer Sb_2_S_3_ nanosheets were embedded into PVA host matrix to fabricate flexible SA film with uniform thickness and distribution compared with an aqueous solution. The whole preparation process was displayed as the flow diagram in Figure 1. Firstly, the bulk Sb_2_S_3_ was grinded into black powder by a mortar for 10 min, so that the particles of Sb_2_S_3_ were as small as possible in the size of micron or nanoscale. Next, 45 mg of Sb_2_S_3_ powder was added into 5 mL of 30% alcohol, followed by ultrasonication with an ultrasonic cleaner (JP-020) at 30 °C for 8 h to mix the compounds evenly. By centrifugation with a centrifugal machine (TGL-16, 5 mL × 8) at 2000 rpm for 30 min, 90% supernatant was pipetted as the final Sb_2_S_3_ solution. Then, 2 mL of Sb_2_S_3_ solution and 2 mL of 1 wt% PVA solution were blended, followed by continuous sonication for 8 h. After centrifuging, the uniform black Sb_2_S_3_-PVA dispersion solution was obtained. The Sb_2_S_3_-PVA films were formed on flat substrates by spin coating and then were parched in an oven at 30 °C for 4 h. Finally, the fiber-compatible SA was assembled by sandwiching a 0.8 × 0.8 mm^2^ Sb_2_S_3_-PVA film between end faces of fiber jumpers with a flange.

### 2.2. Characterization of Sb*_2_*S*_3_* and Sb*_2_*S*_3_*-PVA Film

**Figure 2:**
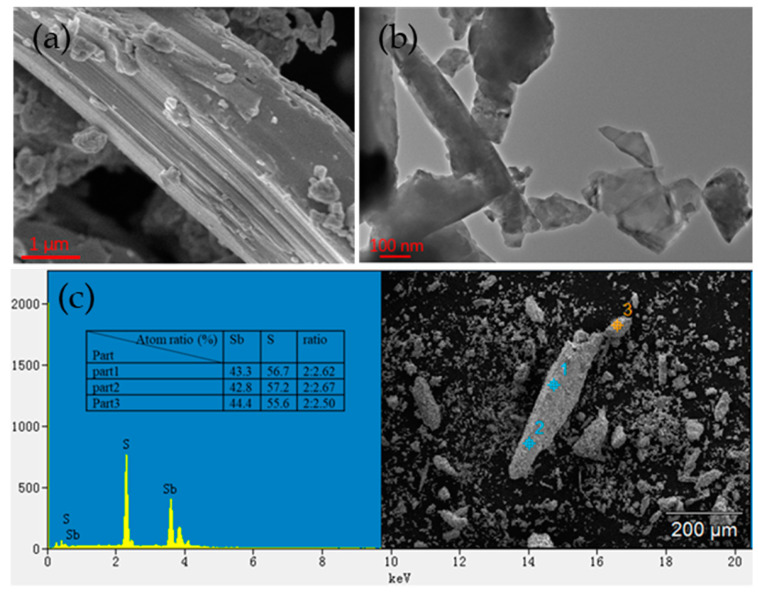

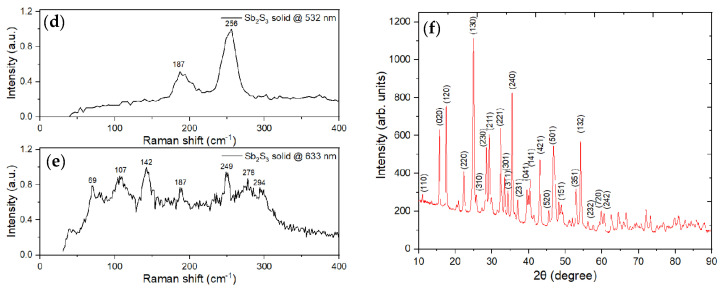
Characterization of Sb_2_S_3_ solid: (**a**) SEM image; (**b**) TEM image; (**c**) EDS spectrum; (**d**,**e**) Raman spectra at 532 and 633 nm; (**f**) XRD pattern.

Various structural and optical properties of Sb_2_S_3_ solid and Sb_2_S_3_-PVA film were explored meticulously. The Sb_2_S_3_ solid was detected first. The microstructures of the Sb_2_S_3_ solid were recorded by a scanning electron microscope (SEM, JSM-7610F) with a magnification of 20,000 times and a transmission electron microscope (TEM) with an optical resolution of 100 nm; the corresponding SEM and TEM images are shown in Figure 2a,b. The SEM image exhibited the layered structures of nanosheets, while the TEM image demonstrated fine stratification of particles through further amplification, indicating that the Sb_2_S_3_ nanosheets with a high crystallinity and well-layered structure were prepared in our work. To explore the elemental composition of Sb_2_S_3_, energy dispersion spectroscopy (EDS) spectra were tested for three times at different parts of Sb_2_S_3_ solid in order to reduce measurement error, as seen in the inset in Figure 2c. As for the three parts of the Sb_2_S_3_ particle, there was no significant difference in their corresponding EDS spectra, revealing uniform distribution of different elements in our prepared Sb_2_S_3_ solid at different parts, so only the EDS spectra of part 1 was shown in Figure 2c. There were only peaks of Sb and S, with an element ratio of about 2:2.6 for the three parts of the particle, which was approximately consistent with the chemical formula of Sb_2_S_3_. Figure 2d,e showed Raman shift spectra of Sb_2_S_3_ solid at 532 and 633 nm, respectively. As shown in Figure 2d, characteristic B_1g_ Raman modes are located at 187 and 256 cm^−1^ under test wavelengths of 532 nm, the position of peaks matched well with the typical frequency shift of Sb_2_S_3_ in Ref. [39]. In Figure 2e, when the test wavelength was changed into 633 nm, Raman shift peaks appeared at 142, 187, 249, 278 and 294 cm^−1^, approximatively in agreement with the peaks in the previous literature [40]. According to the difference between Figure 2d,e, Raman shift peaks would change with the variation of test wavelengths, and similar conclusions have been reported in ref. [41]. As depicted in the XRD pattern in Figure 2f, there were strong and sharp peaks in accord with the peaks of Sb_2_S_3_ in ref. [40], suggesting that the Sb_2_S_3_ powder prepared in our work possessed high crystallinity and purity.

**Figure 3:**
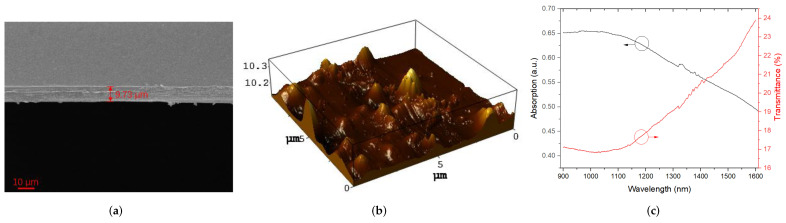
Characterization of Sb_2_S_3_-PVA film: (**a**) SEM image of thickness; (**b**) AFM image of surface; (**c**) Linear absorption and transmittance curves.

Subsequently, the Sb_2_S_3_-PVA film was tested carefully. As seen in the SEM image and atomic force microscope (AFM) image displayed in Figure 3a,b, the Sb_2_S_3_-PVA film with thickness of about 10 μm and a surface roughness within 0.1 μm were recorded by SEM and AFM, respectively, revealing that a relatively uniform and flat Sb_2_S_3_-PVA film was prepared in our work. The absorption and transmittance of Sb_2_S_3_-PVA film coated on quartz glass substrates were measured by a UV-3600 spectrophotometer in wavelength range from 900 to 1600 nm; corresponding absorption and transmittance curves were depicted in Figure 3c. The transmittance of Sb_2_S_3_-PVA film ranges from 24% to 17% as wavelength decreased from 1600 to 900 nm, ending up with the saturation of about 17% due to the nonlinear saturable absorption effect. In addition, there was fairly strong absorption near 1560 nm, indicating that Sb_2_S_3_-PVA film had promising potential as a saturated absorber in pulsed Er-doped fiber laser.

**Figure 4:**
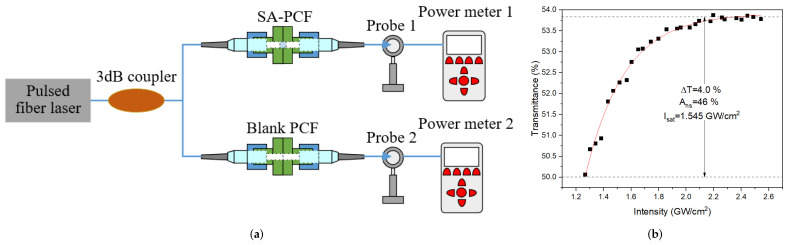
(**a**) Experimental setup for nonlinear absorption measurement of Sb_2_S_3_-PVA SA; (**b**) nonlinear absorption curve of Sb_2_S_3_-PVA SA.

The nonlinear absorption of Sb_2_S_3_-PVA SA was measured by power-dependent transmission scheme based on a home-made 1550 nm NPR pulsed fiber laser with pulse width of 268 fs and a repetition rate of 57 MHz, corresponding measurement setup of which is displayed in Figure 4a. The average output power of fs pulses could range from 24 to 80 mW by adjusting current expediently, so that there was no need to place an attenuator. The fs pulses via a 3 dB coupler were separated into two paths, the reference path and sample measurement path. The reference light via the blank PCF was directly measured by the probe 2 of optical power meter 2, while the measurement light via Sb_2_S_3_-PVA SA was recorded by the probe 1 of optical power meter 1. The nonlinear transmittance curve was fitted with the formula:

(1) $$T\left(I\right)=1-\Delta T\times \exp(-I/I_{sat})-A_{ns}$$

where T was transmittance, ΔT was modulation depth, I was intensity, $I_{sat}$ was saturable intensity, and $A_{ns}$ was non-saturable absorbance. The corresponding fitting curve was described in Figure 4b. According to the graph, the modulation depth, saturable intensity and non-saturable absorbance were 4.0%, 1.545 GW/cm^2^ and 46%, respectively, demonstrating that Sb_2_S_3_ was a promising candidate as a saturable absorber. 

## 3. Experimental Setup

**Figure 5:**
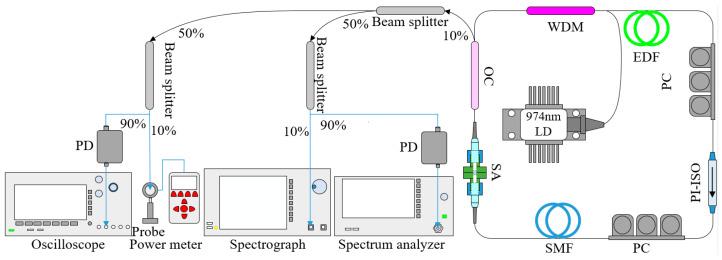
Experimental setup of the fiber laser based on a Sb_2_S_3_-PVA SA.

The experimental setup of the fiber laser based on a Sb_2_S_3_-PVA SA was demonstrated in Figure 5. A 470 mW 974 nm laser diode (LD, FL-974-500-B) with peak wavelength of ~974.7 nm and 3 dB spectrum bandwidth of ~0.16 nm was used as the pump source. The pump light was delivered into the ring cavity via a 980/1550 nm wavelength division multiplexer (WDM). A segment of 10 m MP980 erbium-doped fiber (EDF, −20 ps/nm/km) was employed as the laser gain medium. Two polarization controllers (PCs) were used to adjust the polarization states in the ring cavity while a polarization independent isolator (PI-ISO) was applied to guarantee the unidirectional propagation. Except 10 m pigtails of fiber components, a segment of 35 m single mode fiber (SMF, 17 ps/nm/km) was inserted to optimize the parameters in the cavity, thus the total length of fiber cavity was about 55 m, corresponding total dispersion of fiber cavity and theoretical value of fundamental frequency were about −0.72 ps^2^ and 3.76 MHz, respectively. The compatible SA was assembled by clamping Sb_2_S_3_-PVA film between two fiber jumpers with a flange, so that it could be integrated into the all-fiber laser. The 10% output pulses via a 10:90 optical coupler (OC) was divided by a 50:50 beam splitter. Subsequently, both output ports were connected to the same 10:90 OCs in order to divide output pulses into four paths, so that pulse waveforms, optical spectra, radio frequency (RF) spectra and average power of output pulses could be monitored simultaneously. Both of the 90% output ports were connected to two high-speed positive-intrinsic-negative (PIN) photodetectors (PD 03, 3 GHz bandwidth, 1020–1650 nm) in order to convert optical signals into electrical signals. Then these signals could be displayed as pulse waveforms by a digital phosphor oscilloscope (Tektronix DPO4104B, 1 GHz bandwidth, 5 G samples/s) or recorded as RF spectra by a real-time spectrum analyzer (ROHDE&SCHWARZ, FPC1000). The other 10% output ports could be shown as optical spectra by an optical spectrum analyzer (MS9740B, 0.6–1.75 μm) or recorded as average output power by an optical power meter (OPHIR, NOVA II) with a probe (OPHIR, PD300-IR).

## 4. Experimental Results and Discussion

**Figure 6:**
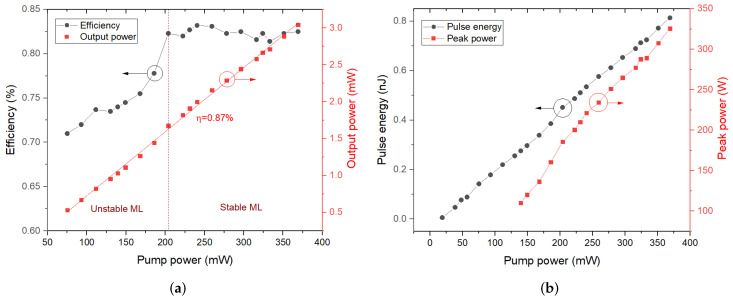
(**a**) Relationship between pump powers with output power and optical-to-optical conversion efficiency; (**b**) Relationship between pump powers with peak power and pulse energy for mode-locked pulses.

In this experiment, firstly, without inserting the SA into fiber laser cavity, only continuous wave was detected whatever we adjusted pump power and PCs. Subsequently, with SA inserting into the laser cavity, mode-locked pulses appeared when pump power reached the threshold of 75.1 mW. As pump power increased from 75.1 to 369 mW, mode-locked pulses could always be observable, and possessed average power, increasing from 0.53 to 3.04 mW, with a slope efficiency of 0.87%, The corresponding relationship curves between pump powers with output power and optical-to-optical conversion efficiency for mode-locking are shown in Figure 6a. When pump power was below 200.3 mW, there were unstable mode-locked pulses with constant increases of optical-to-optical conversion efficiency, while the mode-locking was stable under the pump power over 200.3 mW with fairly stable optical-to-optical conversion efficiency of about 0.82%. Figure 6b demonstrates the relationship between pump powers with peak power and pulse energy for mode-locked pulses. Single pulse energy grew from 0.14 to 0.81 nJ as pump power increased from 75.1 to 369 mW, while peak power increased from 110.1 to 325.6 W with the increase of pump power from 139.4 to 369 mW, because no autocorrelation traces of mode-locked pulses were observed below the pump power of 139.4 mW. However, when the pump power exceeded 369 mW, the fundamental frequency mode-locked pulses disappeared, while harmonic mode-locked operations continued to rise. When pump power was reduced under 369 mW, the stable mode-locked operation began to recover again. These procedures were repeated for several times, and similar phenomena could always be observed, revealing that the SA suffered no damage. In our opinion, the mode-locked pulses were split into harmonics with the increase of pump power, because of the peak power-limiting effect based on the soliton area theorem [42].

**Figure 7:**
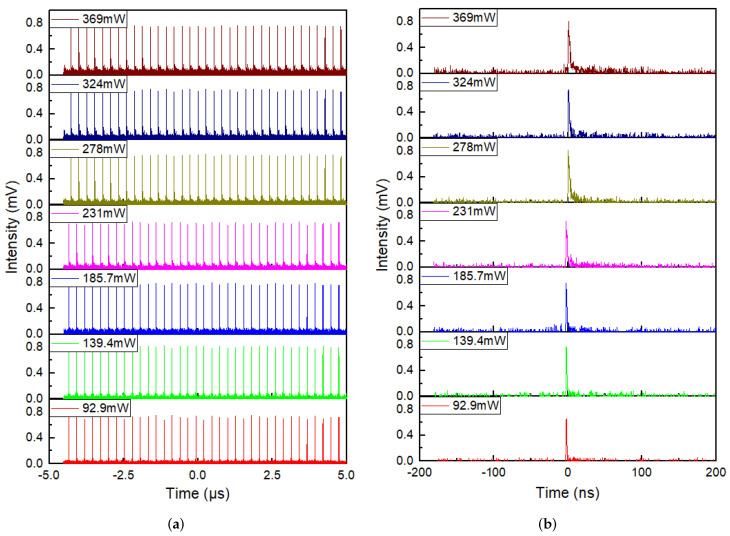
(**a**) Waveforms; (**b**) Single pulse profiles of mode-locked pulses at different pump power.

**Figure 8:**
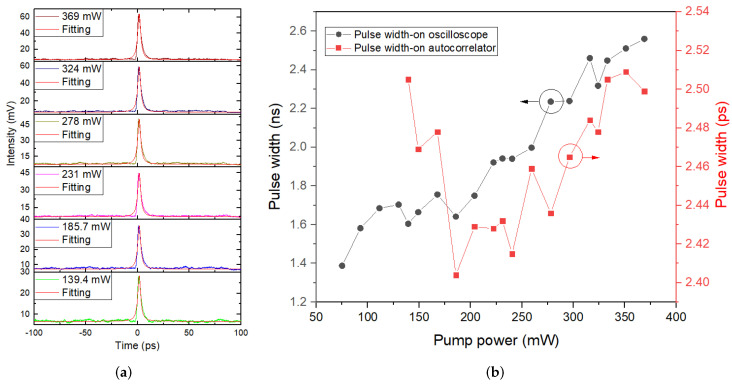
(**a**) Autocorrelation traces of mode-locked pulses at different pump powers; (**b**) Relationship between pulse width shown on both oscilloscope and autocorrelator with pump power for mode-locking.

The waveforms and corresponding single pulse profiles of mode-locked pulses at different pump powers were shown in Figure 7a,b. Although there was weak noise increasing as pump power enhanced in the waveforms and the corresponding single pulse profiles, the pulse trains possessed almost the same height and scarce fluctuation at any pump power, as well as the single pulse shapes were clean with ideal shapes, revealing that mode-locked operations of the fiber laser were stable. In addition, it was obvious that except for pulse intensity enhanced with the increase of pump power, the pulse width displayed on the oscilloscope broadened from 1.39 to 2.56 ns when pump power increased from 75.1 to 369 mW; detailed relationship curves between pulse width with output power are displayed in Figure 8b. Due to the finite bandwidth of both photodetector (3 GHz) and oscilloscope (1 GHz), there were fairly large errors of the pulse width values displayed on the oscilloscope. In order to demonstrate real and effective pulse width values, the autocorrelation traces of mode-locked pulses were observed by an autocorrelator (FR-103XL) at different pump powers. Corresponding autocorrelation traces and real pulse widths at different pump powers are displayed in Figure 8a,b, respectively. By the way, pulse widths were the real values calculated by multiplying autocorrelation signal widths by a scale factor of 0.648. The autocorrelation signals with nice and regular shapes agreed with sech^2^ fitting curves very well. Their intensity heightened as the pump power rose, while pulse widths decreased firstly and then increased when the pump power increased, with a minimum value of 2.4 ps under 185.7 mW and a variation range of less than 0.1 ps. However, there were no autocorrelation traces of mode-locked pulses recorded below the pump power of 139.4 mW. In our opinion, at the beginning of mode-locking, there were unstable mode-locked operations at first with such low output power and such broad pulse widths, that the mode-locked pulses were out of the measurement range of the autocorrelator, resulting in no autocorrelation signals. As mode-locking became gradually stable, pulse widths gradually decreased to a minimum value of 2.4 ps. However, the mode-locking became unstable when the pump power was too high, so that the pulse width increased again. The fairly small variation range of pulse width revealed that mode-locking was relatively stable throughout the whole range of pump power.

**Figure 9:**
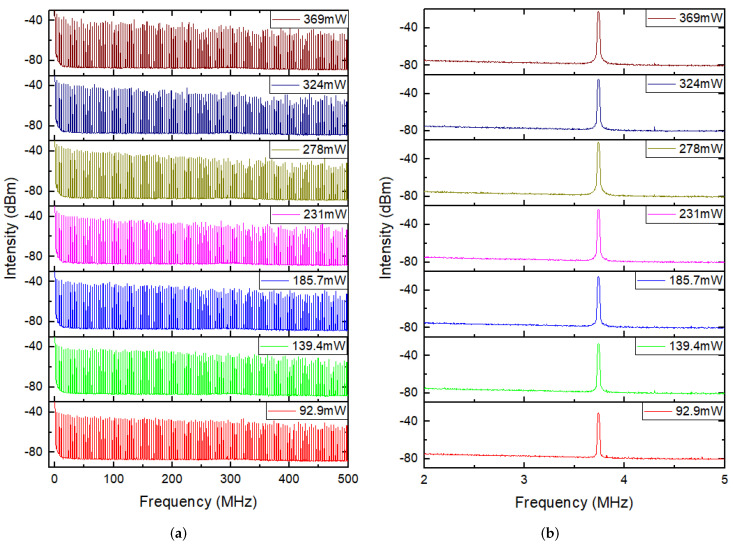
(**a**) RF spectra in the range of 500 MHz; (**b**) Single peak frequency in the range of 3 MHz of mode-locked pulses at different pump powers.

**Figure 10:**
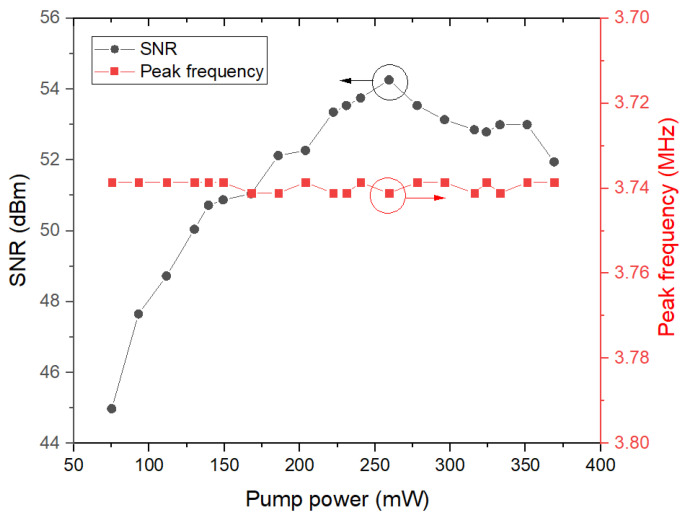
Relationship between pump powers with peak frequency and SNR for mode-locking.

RF spectra and single peak frequency of mode-locked pulses in the range of 500 MHz and 3 MHz with a resolution bandwidth (RBW) of 10 kHz and video bandwidth (VBW) of 100 Hz at different pump powers are demonstrated in Figure 9a,b, respectively. In Figure 9a, there was a flattening off and then a slight decline in frequency combs as the frequency range expanded at any pump power. However, there were slight fluctuations at pump powers below 278 mW, while relatively violent fluctuations were in the RF spectra with pump powers over 278 mW, suggesting the descending stability of mode-locking in high pump powers. Figure 10 demonstrated detailed relationship curves between pump powers with peak frequency and SNR for mode-locking; in agreement with the Figure 8b, there was the same peak frequency of about 3.74 MHz and 3 dB bandwidth of about 10 kHz at any pump power. In Figure 10, peak frequency was almost the same (about 3.74 MHz) with the variation within 2.5 kHz, possibly due to recording or sampling errors. However, the signal to noise ratio (SNR) rose from 45 to 54.3 dB under pump power from 75.1 to 259.3 mW, while fell down to about 52 dB as pump power continued growing up to 369 mW, suggesting that mode-locked pulses perhaps possessed maximal SNR and the most stable operation at pump power of about 259.3 mW. In other words, the stability of mode-locking would degrade over a pump power of about 260 mW, which is in accord with the fluctuation variation of RF spectra in Figure 9a.

**Figure 11:**
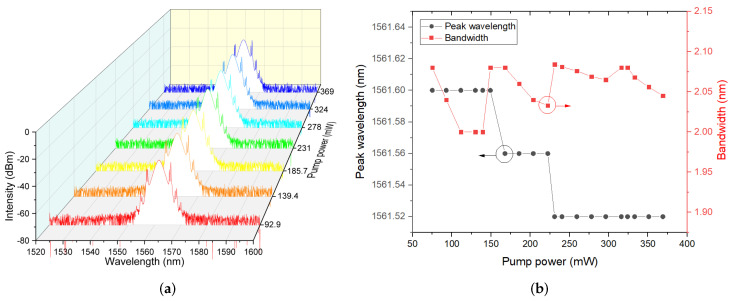
(**a**) Optical spectra of mode-locked pulses at different pump powers; (**b**) Relationship between pump powers with peak wavelength and 3 dB bandwidth for mode-locking.

The optical spectra of mode-locked pulses at different pump powers is shown in Figure 11a. There are obvious Kelly sidebands in the optical spectra, which is in agreement with the anomalous dispersion regime. Figure 11b displays the relationship curves between pump powers with peak wavelength and 3 dB bandwidth for mode-locking. The peak wavelength was blue-shifted from 1561.6 to 1561.52 nm as pump power increased from 75.1 to 369 mW. In detail, there were three stable ranges of pump power: (75.1–149.1 mW), (167.8–222.3 mW) and (231–369 mW), with corresponding fixed peak wavelengths of 1561.6, 1561.56 and 1561.52 nm, respectively. Peak wavelengths were blue-shifted from 1561.6 to 1561.56 nm under pump power from 149.1 to 167.8 mW and from 1561.56 to 1561.52 nm under pump power from 222.3 to 231 mW, respectively. In every stable pump power range, the 3 dB bandwidth collapsed with the increase of pump power, which is in agreement with the opposite variation trend of pulse width, which enlarged gradually as the pump power increased. In our opinion, we thought that the saturable absorption and optical properties of Sb_2_S_3_-PVA SA may be responsible for the blue shift. However, the time-bandwidth product (TBP) was calculated to be about 0.629 at pump power of 369 mW, which was slightly higher than the theoretical limit value of 0.315, indicating that optical pulses were little chirped, which was mainly attributed to the large dispersion value of −0.72 ps^2^ produced by the long length of the laser cavity. Thus, the parameters of this fiber cavity should be further optimized in our next experiment.

In addition, insert loss of the Sb_2_S_3_-PVA SA was measured for about 1.6 dB based on a transmittance method, while there was no obvious polarization-dependent loss, indicating that the low output power and slope efficiency mainly derived from the large insert loss produced by the SA. Furthermore, the stability of the mode-locked Er-doped all-fiber laser was tested. The stable mode-locked operation could sustain under pump power of 369 mW for more than 30 min, demonstrating the durability of the prepared Sb_2_S_3_-PVA SA. There were only continuous wave and no mode-locked pulses once the SA was removed from the laser cavity, suggesting that the mode-locked operations derived from the saturable absorption effect of Sb_2_S_3_-PVA SA.

For comparison, the performance of the mode-locked EDF laser demonstrated in this work with those reported based on other 2D-materials SAs is summarized in Table 1. Compared to other 2D materials, there were the highest average power and the largest single pulse energy in this work. In addition, pulse width, modulation depth and the SNR obtained in this work were basically at an intermediate level compared with corresponding values among other 2D-materials-SA mode-locked EDF lasers according to Table 1. In summary, our experimental results suggested that Sb_2_S_3_ was indeed a promising SA candidate to obtain mode-locked pulses in ultrafast fiber lasers.

## 5. Conclusions

In conclusion, Sb_2_S_3_ was successfully prepared with the liquid phase exfoliation method and embedded into PVA as an SA in the passively mode-locked Er-doped fiber laser for the first time. Stable mode-locked pulses with frequencies of about 3.74 MHz could be obtained as the pump power increased from 75.1 to 369 mW with a slope efficiency of about 0.87%. The maximum average output power of 3.04 mW and maximum peak power of 325.6 W were accomplished at a pump power of 369 mW. The SNRSs were always greater than 45 db. There was a maximum SNR of 54.3 dB at a pump power of about 260 mW, revealing fairly stable mode-locked operations throughout the whole range of pump power all the time. Based on Sb_2_S_3_-PVA SA with a modulation depth of 4.0% and a saturable intensity of 1.545 GW/cm^2^, a minimum pulse width value of 2.4 ps with a variation range less than 0.1 ps was successfully realized, suggesting that Sb_2_S_3_ is indeed a promising SA candidate to obtain both mode-locked pulses and harmonic mode-locked pulses in ultrafast fiber lasers.

## Figures and Tables

**Table 1:** Performance summary of passively mode-locked EDF lasers with 2D-materials-based SAs.

2D Mater.	Integration Method	λ nm	F_rep_ MHz	τ Ps	P_ave_ mW	E nJ	ΔT %	SNR dB	Ref.
Graphene	Sandwiched	1576	10.9	3.2	3.02	-	-	40	[8]
Graphene	Microfiber	1531.3	1.89	1.21	0.45	-	4.37	60	[9]
GO	Microfiber	1560	7.47	18	1.2	-	5.75	70	[10]
BP	Sandwiched	1561	5.268	1.438	-	-	0.8	60	[14]
Bi_2_Se_3_	Sandwiched	1557.5	12.5	0.66	1.8	-	3.9	55	[15]
Bi_2_Te_3_	Sandwiched	1557	8.635	1.08	0.25	-	2.0	60	[16]
Sb_2_Te_3_	Sandwiched	1558.6	4.75	1.8	0.5	0.105	-	60	[17]
MoS_2_	SPF	1568	26.02	4.98	-	0.08	2.5	63	[19]
MoSe_2_	Sandwiched	1558.25	8.028	1.45	0.44	0.0548	0.63	61.5	[20]
WS_2_	SMPCF	1563.8	19.57	0.808	2.64	0.1336	3.53	60.5	[21]
TiS_2_	Microfiber	1563.3	22.7	0.812	-	0.0253	8.3	60	[22]
PtS_2_	Sandwiched	1572	15.04	2.064	1.1	-	7.0	43	[23]
PtSe_2_	Microfiber	1563	23.3	1.02	-	0.53	4.9	61	[24]
ReS_2_	Sandwiched	1558.6	5.48	1.6	0.4	-	0.12	-	[30]
Sb_2_S_3_	Sandwiched	1561.6	3.74	2.4	3.04	0.81	4.0	54.3	This work

SPF: side-polished fiber; SMPCF: single mode photonic crystal fiber; λ: central wavelength; F_rep_: repetition frequency; τ: pulse width; E: single pulse energy; ∆T: modulation depth.

## Data Availability

Data are contained within this article.

## Sample Availability

Samples of the compounds are not available from the authors.
